# How do hospital-based nurses and physicians identify the palliative phase in their patients and what difficulties exist? A qualitative interview study

**DOI:** 10.1186/s12904-019-0439-0

**Published:** 2019-07-09

**Authors:** Isabelle Flierman, Ineke C. Nugteren, Rosanne van Seben, Bianca M. Buurman, Dick L. Willems

**Affiliations:** 1 Department of General Practice, Section of Medical Ethics, Amsterdam Public Health Research Institute, Amsterdam UMC, Univeristy of Amsterdam, Amsterdam, The Netherlands; 2 Department of Internal Medicine, Section of Geriatric Medicine, Amsterdam Public Health Research Institute, Amsterdam UMC, University of Amsterdam, Amsterdam, The Netherlands; 3 ACHIEVE - Centre of Applied Research, Faculty of Health, Amsterdam University of Applied Sciences, Amsterdam, The Netherlands

**Keywords:** Palliative care, Identification, Hospitals, Physicians, Nurses

## Abstract

**Background:**

Early start of palliative care improves the quality of life of eligible patients and their relatives. However, in hospital, patients who could benefit from palliative care are often not identified timely. The aim of this study is to assess how hospital-based nurses and physicians define the palliative phase, how they identify the palliative phase and what difficulties they face.

**Methods:**

Semi-structured interviews were held with ten nurses and 18 physicians working at seven hospitals in the Netherlands. Data was analysed using thematic analysis.

**Results:**

Nurses and physicians feel insecure about how to define the palliative phase and differentiate between an acute and extended phase. Great variation existed in what life expectancy is attributed to each phase. A variety of ways to identify the palliative phase were described: 1) Prognostication. 2) Treatment trade-off. 3) Assessment of patients’ preferences and needs. 4) Interprofessional collaboration. Professionals base prognostication on their experience but also search for clinical indicators. When benefits of treatment no longer outweigh the negatives, this was considered an, albeit late, identification point. To start a conversation on a patients’ palliative care needs was found to be difficult. Therefore, some respondents wait for patients to vocalize preferences themselves. Many professionals rely on interprofessional collaboration for identification, however uncertainty exist about responsibilities. Difficulties in identification occurred because of variance in definitions, unpredictability of non-oncological diseases, focus on treatment and difficulties in communication and collaboration.

**Conclusion:**

These results provide insight into the challenges and difficulties hospital-based professionals experience in timely identification of patients with palliative care needs.

**Electronic supplementary material:**

The online version of this article (10.1186/s12904-019-0439-0) contains supplementary material, which is available to authorized users.

## Background

Historically, palliative care has been associated with patients that are dying. However, as stated in the WHO definition, palliative care should be integrated earlier in a patient’s disease trajectory, because this can improve quality of life, reduce symptom burden, and leads to less aggressive treatments and fewer hospitalizations [1–4]**.** Yet, in daily practice, physicians and nurses often do not initiate palliative care until death is imminent—if they initiate it at all [5–7]. A challenge for timely initiation is the difficulty in identifying which patients are eligible for palliative care, especially because more people are now dying from chronic progressive diseases that often follow less predictable disease trajectories [8, 9].

The majority of people with end-stage disease require acute hospital care, due to increased symptom burden, in the last years of life and many elderly in particular, die shortly after an acute hospitalization [10–13]. Hospital-based professionals are therefore well positioned to identify patients who could require palliative care [14]. However, thus far, most studies on identification of patients in the palliative phase have focused on general practitioners’ (GP) perspectives and not hospital professionals, because patients often prefer home as the place of care and death [15–17]. Furthermore, nurses, who often work closer to patients, could be important in assessing which patients are in need of palliative care, although little is known about their role.

Dalgaard et al., [18] described different methods for early identification within the hospital: prognostication, assessment of care needs and use of identification ‘instruments’, such as the ‘Surprise Question’ (would I be surprised if the patient were to die in the next year?) [19] and instruments that score on clinical and disease-related markers [20, 21]. The authors found that for none of the methods, there was sufficient evidence to recommend routine use in clinical practise.

In order to improve identification within the hospital setting and overcome existing barriers, we need to better understand what the current manners are for identification and what difficulties exist. Therefore, the aim of this study is threefold. 1) to explore how physicians and nurses working in the hospital define the palliative phase 2) how professionals identify the palliative phase in their patients in daily practice 3) what are perceived barriers to identifying the palliative phase in daily practice.

## Methods

### Study design

To provide an in-depth understanding of hospital-based physicians’ and nurses’ experiences with and perspectives on identification of the palliative phase within the hospital setting, a phenomenological approach was chosen [22, 23], consisting of semi-structured interviews, which is a method particularly suitable to gain a comprehensive insight into experiences and perspectives [22, 23]. The interviews were held between September 2016 and 2017. We followed the consolidated criteria for reporting qualitative research (COREQ) guidelines [24] (Additional file 1).

### Study participants and recruitment

We recruited nurses, residents and medical specialists from the departments of internal medicine, oncology, geriatrics, cardiology, nephrology and pulmonology from one academic and six general hospitals. Professionals within these specialties often care for patients in the palliative phase and consequently were expected to have experience with identification. We recruited both nurses and physicians because of their different perspectives. We recruited residents, who provide most of the daily care for hospitalized patients, and specialists, who additionally see patients at the outpatient clinics. We recruited nurses with general training and those with additional specialty training, for example, in heart failure.

Participants were purposefully sampled based on specialization, hospital type, work experience and experience in palliative care. Six people declined participation because of time restraints. We recruited participants via email through the professional network of the researchers and through snowball sampling. The invitation mentioned the goal of the interview.

### Data collection

The first author (IF), a physician and PhD student with training in qualitative research, conducted all interviews. Interviews were one-on-one and conducted in Dutch at participants’ workplace, and in two cases at a library. Notes were made during each interview, which were used to make a summary of the interview which was sent to participants, and to provide context for the analysis. The interviews were guided by a topic list with open-ended questions and probing questions. The first and last authors (IF and DW) created the topic list based on previous research on this topic [15, 16, 18]. The other authors critically reviewed the topics. Two pilot interviews were held, after which the authors critically reviewed the topic list and adjusted the questions accordingly. The topic list can be found in Additional file 2. We obtained written, informed consent, and audio recorded and transcribed verbatim all interviews.

### Analysis

Data was analysed thematically, a method for identifying, analysing and reporting patterns, i.e. themes, within the data [25]. An initial ‘open-coding’ scheme was chosen because we aimed for data-driven analysis and an broader understanding of identification of the palliative phase in daily practice. IF read and reread all transcripts to become familiarized with the data. IF and IN coded the initial five transcripts independently with an ‘open-coding‘scheme (inductive coding), however some codes resulted specifically from the questions asked for example “Are there differences in identification for different diseases?” and were therefor the result of deductive coding. IF and IN discussed differences in coding until consensus was reached, if difference persisted a third researcher (DW) was consulted. After the initial open-coding scheme, IF and IN created an initial codebook, which IF used to code the remaining transcripts. This codebook was however not static, if necessary new codes emerged from the data these were added. After initial analysis, IF sorted the different codes into potential themes and subthemes. In the next phase, IF reread all the coded data and assessed the appropriateness of the formulated themes and, if necessary, adjusted, added or removed themes. During the analysis and writing process, the results were repeatedly discussed with all of the authors. Coding and analysis was done using the MAXQDA software program [26]. Data saturation was reached, because the last five interviews revealed no new concepts and themes [27].

## Results

We conducted 10 interviews with nurses, 12 with specialists, and six with residents. Table 1 presents a summary of their characteristics. The interviews lasted between 26 and 68 min with an average of 49 min.

**Table 1:** Participants characteristics

Respondent	Gender	Age	Department	Hospital (centre)	Years of work experience	(work) Experience/training in palliative care
N1	F	30–39	Nurse (internal medicine)	University hospital (1)	7	
N2	F	40–49	Nurse (internal medicine/oncology)	University hospital (1)	19	
N3	F	20–29	Nurse (pulmonology)	University hospital (1)	4	
N4	F	20–29	Nurse (cardiology)	University hospital (1)	5	Extracurricular courses
N5	M	50–59	Nurse practitioner (cardiology)	University hospital (1)	37	Course on end of life communication
N6	F	30–39	Nurse (palliative care team)	General hospital (5)	22	Palliative care team member, specialist training
N7	F	40–49	Nurse (pulmonology)	General hospital (5)	17	Extracurricular course
N8	F	30–39	Nurse (internal medicine)	University hospital (1)	2	
N9	F	20–29	Nurse (internal medicine)	University hospital (1)	3	
N10	F	20–29	Nurse (internal medicine)	University hospital (1)	1.5	
R1	M	30–39	Resident (internal medicine)	University hospital (1)	2.5	
R2	F	30–39	Resident (internal medicine)	General hospital (2)	0.5	
R3	M	30–39	Resident (cardiology)	University hospital (1)	6	Extracurricular training
R4	F	30–39	Resident (nephrology)	University hospital (1)	6	
R5	M	20–29	Resident (cardiology)	General hospital (4)	0.5	
R6	F	20–29	Resident (geriatrician)	General hospital (6)	2	
P1	F	40–49	Oncologist	General hospital (2)	12	Palliative care team member
P2	M	50–59	Oncologist	General hospital (6)	14	Extracurricular courses
P3	M	40–49	Geriatrician	General hospital (6)	19	Worked in palliative care unit
P4	F	50–59	Nephrologist	University hospital (1)	30	
P5	M	40–49	Geriatrician	General hospital (5)	17	Palliative care team member, extracurricular courses
P6	M	60–70	Pulmonologist	General hospital (5)	31	Palliative care team member, specialist training
P7	M	60–70	Internist	General hospital (3)	31	
P8	F	40–49	Pulmonologist	General hospital (7)	11	Palliative care team member, specialist training
P9	F	40–49	Cardiologist	University hospital (1)	12	
P10	M	50–59	Nephrologist	General hospital (4)	25	
P11	F	40–49	Cardiologist	University hospital (1)	9	
P12	M	50–59	Internist/Geriatrician	General hospital (3)	25	Educator in palliative care

The first main topic we addressed was ‘defining the palliative phase’. Respondents described a variety of often intertwining ways to identify the palliative phase in their daily practise, leading to four other main themes: (1) prognostication, (2) treatment trade-off and (3) patients’ preferences and needs (4) interprofessional collaboration and responsibilities. Specific barriers and facilitators to identification emerged within each of these themes.

### Defining the palliative phase

The majority of respondents have difficulty—and feel insecure about—defining which patients they consider to be in the palliative phase. Respondents often distinguished between an ‘acute’ palliative phase, in which death is imminent, and a more ‘extended’ phase, in which patients have a (potentially) life-threating disease but are not yet dying:

“*You have of course the terminal phase, where you expect the patient to die within the foreseeable future. That is, then you are very active with providing palliative care. But palliation, in my opinion, can also mean you are not providing terminal care, but you are active with the end of life.”*(P3 geriatrician)

Many considered the moment focus completely switches from curative care to symptom control and improving quality of life as the starting point of the palliative phase. Some believed thinking about the palliative phase was only useful when it had clear consequences such as withdrawal of treatment, discharging a patient to primary care or consulting the specialist palliative care team. The ‘acute’ palliative phase, also frequently described as the terminal phase or dying phase, was consequently clearer defined for respondents because the focus was fully on comfort and resulted in treatment withdrawal or palliative sedation. Respondents defined the extended phase differently. Descriptions frequently used were the moment when there were no curative options left, or when patients had a limited life expectancy. However, what was considered a limited life-expectancy ranged from weeks to years. Where many nurses, cardiologists and nephrologists spoke of a shorter life expectancy, geriatricians and oncologists more often spoke of a life expectancy of years.

*“How long the foreseeable future is, that is complicated. When you know that death because of the underlying disease is certain, but yes that is difficult. Some patients will die somewhere between now and 30 years. That of course of is not really the palliative phase. Let’s say the last months. Yeah maybe shorter. I don’t know.” (R5, resident cardiology)*

#### Prognostication

Assessing the prognosis of a patient is an important step in identification for respondents. Respondents often found prognostication easiest in cancer patients, where metastasized disease means a clear transition point, and respondents can base their assessment on ‘hard measurements’ such as scans. On the other hand the unpredictable course of organ failure and dementia resulted in difficulty prognosticating:

*“Look, the difference between, for example, the palliative phase in an oncological patient and the palliative phase in a heart failure patient . . . if you have an untreatable metastasized lung carcinoma, then you’ll die. That is, that is certain. And with heart failure, you know you’ll die earlier, but you don’t know when.”* (N5, nurse practitioner cardiology).

For prognostication, respondents often rely heavily on their ‘clinical glance’, a term Dutch health care providers often use for an intuitive assessment of the severity of a patient’s situation. Respondents had difficulty explaining this ‘clinical glance’ but said it was mainly based on previous experiences and physical or psychological signals from patients. Nurses often described it as a discomfort in following certain treatment orders:

*“It is often nurses who already experience a sort of feeling in their stomach, a sort of feeling like ‘what are we actually doing?’ If you have the feeling like ‘what are we actually doing’ it is a sign that something is up.”* (N8, nurse internal medicine department).

Inexperience with certain diseases resulted in insecurity in respondents to trust their gut feeling. Furthermore, when respondents had experiences with cases that did not follow the course they had expected, this could make respondents doubt their ‘clinical glance’:

*“There are no good prediction models. It’s more a like an individual clinical glance. What have you seen before? And you should always be careful with going with your own gut feeling and your own experience, because your own experience does not have to match with the patient that is sitting in front of you, or laying, or panting.”* (R3, resident cardiology).

Respondents try to objectify their gut feeling, by looking for clinical indicators. Different clinical indicators were mentioned that could trigger identification: general, disease specific, physical, and psychological. One such general indicator was recurrent hospitalizations. Respondents describe that physical indicators can be very clear, such as weight loss or a change in functional status, but can also be subtle. Some respondents also mentioned psychological signals such as fear, depression and decline in cognitive functioning. Sometimes they observe resignation, as described here by a nurse practitioner:

*“But in the end, people themselves see that there is no more hope. . . . And then you eventually see somebody gives up. . . . You see that the light in their eyes dampens, and you see that because you know the person so well. . . . So the moment you see that, you know what the situation is, even though you don’t have any numbers.”* (N5, nurse practitioner cardiology).

Respondents view having a longer relationship with a patient as facilitating for prognostication, because it allows them to observe a decline over time. However, some felt having a bond with a patient could prevent them from seeing that a patient is nearing the end of life. A respondent described this situation in caring for a younger patient:

*“I think, however, that that is a pitfall for especially experienced physicians . . . that you get attached to a patient and don’t want to acknowledge that it is going to end soon. And that is something I do think exists.”* (P1 oncologist)

Some respondents were familiar with the ‘Surprise Question’ and use it as a trigger for identification. When asked specifically, respondents said they were unfamiliar with instruments that use lists of general indicators/signals. They felt identification instruments are helpful in creating awareness. However, they questioned the prognostic accuracy, and thought identification of the palliative phase is not as simple as ‘checking off’ certain indicators:

*“But one of those checklists, I think you could use them in clinical practice, definitely. But I do think you need to keep in the back of your head that it is not black and white. So . . . a checklist might not be applicable for each patient.”* (R2, resident internal medicine department)

#### Treatment trade-off

Respondents considered lack of curative treatment options to be closely linked to limited prognoses; however, they also described this lack as a separate manner of identification and hence as a separate theme. The moment all curative options were exhausted was considered a clear transition point to the palliative phase. However, respondents thought this distinction was again less clear in patients with chronic diseases, such as organ failure or diabetes:

*“Some people say that you can’t cure COPD, so everything you do is per definition palliative, but I think that’s nonsense. There is a group of people with COPD that are limited by their dyspnea or fear, and those are the people, if you ask me.”* (P8, pulmonologist).

To decide if curative treatment options are viable, physicians described trying to weigh the benefits of treatment with the negatives and aim for an acceptable balance. They felt a shift to the negative marks the palliative phase, yet this shift often occurs late in the disease progression, especially in organ failure:

*“Yes, but then it shouldn’t be that . . . at a certain moment you agree that, well, there are no treatment options left, … she (*a patient with heart failure*) dies within 24 hours. Then you can’t do anything anymore. See, then you are a rather late.”* (R6, resident geriatrics).

While patients’ opinions on treatment continuation are considered an important part of assessing treatment trade-off, some physicians wait for patients themselves to mention they want to quit or not start treatment. Furthermore, physicians describe they need to have tried all treatment options before ‘accepting’ somebody is in the palliative phase. They described not wanting to have failed in exploring all diagnostic and treatment options that are possible:

*“And you don’t want to admit too quickly, so you want to first thoroughly have explored all different options you have before you say ‘there is indeed really nothing we can do anymore’. So I think you should really have a complete picture and have discussed it with everyone before you say ‘we really have considered it, but it’s a bridge to far’.”* (R5, resident cardiology).

Nurses frequently said they felt physicians often focus on treatment possibilities too long which they considered harmful for patients. An explanation given by respondents was that they were trained to focus on treatment and fixing the acute problem a patient presented with to the hospital. To overcome the continued focus on treatment, some physicians described setting a certain time limit in which a patient should have responded to treatment before withdrawal:

*“Like, we are going to improve the nutritional status and we will do this and that and we are going to do everything optimally for two weeks. And if after two weeks it is still getting worse, then we quit.”* (R4, resident nephrology).

#### Patients’ preferences and needs

Respondents also base their assessments of the palliative phase on patients’ needs and preferences. When the above-mentioned clinical indicators or treatments negatively influence the quality of life of a patient, the need for palliative care is clearer, according to respondents:

*“I think that the moment they become very limited in their functioning, and especially when they, because of it, are not having any fun in their lives, that conversations are needed, like ‘what is it you actually want, and how can I assist’. Yes, so how severe do they find their own suffering, and how much is it obstructing their quality of life.”* (P12, geriatrician).

Respondents described the wishes and preferences of patients concerning their future as being important in their assessments. As stated before, some respondents felt that it was up to patients to vocalize wishes and preferences themselves. However, others actively engage in discussions to explore them when, based on other indicators, they believe a patient might be nearing the palliative phase:

*“And she marked that phase herself because she herself indicated that she did not want any medical interventions anymore.”* (P8, pulmonologist).

Respondents, however, described multiple barriers to initiating these conversations which often stem from the uncertainty they experience in prognostication but also because they didn’t think patients wanted to discuss this or felt it was not their responsibility. Respondents also explained that patients can sometimes be inconsistent in expressing their preferences to other colleagues, which makes respondents reluctant to act on those preferences. Nurses often explained that patients tell them they want to discontinue treatment, but the patients withhold this wish from their physicians:

*“And we as nurses are apparently more accessible, I think, because you have the function of a nurse”. But more accessible to share it with us than with a physician, because when the physician comes by and says, “Well, we are going to this and that and tomorrow we will test that’, then they say”, “Yes, off course”. And we walk in half an hour later and then they say, “Yes, actually I don’t want that, I don’t want those tests anymore.”* (N4, nurse cardiology department)

In patients with dementia and frailty, respondents believed conversations about future care are needed before patients become cognitively impaired, and consequently their focus shifts earlier to patients’ preferences and needs. Respondents described searching for objective clinical indicators or relying on relatives to speak for patients who are already cognitively impaired. Relatives are, in general, considered an important source of information. However, some respondents find that relatives sometimes withhold information that could influence how respondents perceive patients’ overall wellbeing:

*“It lies a little in recognition because it is being obscured, because you are not getting all the information and you often see that the functioning is described better . . . Yes, then you don’t hear how bad somebody is functioning on their own.”* (P12, geriatrician).

#### Interprofessional collaboration and responsibilities

Independent of their work experience, respondents mentioned they value discussions with direct colleagues while identifying patients in need of palliative care. Either to check if their assessment is right, and in cases of nurses to get more support before voicing their concerns to physicians.

*“And of course consultation with a colleague, like: ‘Well, this is what I see. Do you see that as well?”* (P11, cardiologist).

Consulting palliative care team members was mentioned as a consequence of identification but hardly as an aid in identification. Also, respondents did not often mention primary care physicians as colleagues with whom they would discuss identification. However physicians did describe coordinating care with the GP when the palliative phase was identified:

*“Well then I tried to assess how this woman functioned at home. Well, the last years everything had become more difficult (…) And , yes her life had become increasingly more restricted. And then I consulted with her GP.”* (P7, internist)

Many nurses said they are better at identification because they work more closely with the patients. However, some nurses mentioned being hesitant to tell physicians they consider a patient to be in the palliative phase. They do not want to be seen as ‘giving up’ on the patient or that they doubt physicians’ expertise. Physicians, however, mentioned they consider nurses important in signalling and take their opinions seriously:

*“Having the guts to say that you think the treatment or options that we are offering to the patient, well if they are in fact useful? Are we . . . doing the right thing? Well then maybe you are not just undermining the physician’s medical policy. But also, in my own eyes, I also have the feeling that when you say that, I don’t want to help the patient anymore”* (N3, nurse at pulmonology department).

Many nurses and some residents believed identifying patients in the palliative phase is not their responsibility and is up to their superior. However, some specialists themselves, feel it is not their responsibility, for instance when they see patients for a specific problem or an emergency admission:

*“I think that’s a difficult issue. We see more and more that severely ill patients come to the ER, and then it’s not just their acute illness, but everything that was already happening before. Heart failure, the chronic leg ulcer, everything together. And that makes you think ‘yes, should I be the one that all of a sudden, I don’t know you, be the one to say, well actually we are more in the palliative phase.”*(P5, geriatrician)

## Discussion

### Main findings and comparison with other studies

This qualitative study explored how hospital-based physicians and nurses describe the palliative phase, what methods are used to identify the palliative phase and what difficulties exist in daily practise. Identification seems to be a non-structured process that occurs over a longer period of time that consists of prognostication, assessment of treatment trade-offs, assessment of preferences and needs and interprofessional collaboration. Barriers in identification occur because of the variance in definitions that are used, a persistent focus on treatment, the unpredictability of non-oncological diseases, difficulties in communication with patients, uncertainty in responsibility and insufficient interprofessional collaboration.

In 2002 the WHO stated that palliative care should be available for all patients and families facing the problems associated with life-threatening illness and should be initiated early on in disease trajectories [4]. Nonetheless, a first finding of our study was the difficulty and uncertainty our respondents experienced in defining the palliative phase. This finding agrees with previous studies in primary- and secondary-care settings [28, 29], and despite the WHO definition, discussions are ongoing on how we, as researchers and practitioners, should define palliative patients [30]. The purpose of this study was to explore experiences and perspectives of professionals themselves. Therefore, we did not provide respondents with definitions, but instead acquired their own interpretation. Whereas some used definitions of the palliative phase similar to the ‘early palliative care model’, as proposed by Lynn et al., where palliative care starts before all curative options are exhausted [5], many participants associated the palliative phase with the moment all curative options are exhausted or the prognosis is clearly limited. The misunderstanding that the palliative phase is synonymous with the terminal phase is persistent [31]. Consequently, identification will occur late within the hospital setting [18], which prevents patients and their relatives to benefit from early integration of palliative care [1–3].

When and whether identification occurs seems to be highly dependent on a patient’s diagnosis [18]. Whereas prognostication and the weighting of treatment options is a clear transition point to the palliative phase in cancer patients, in non-cancer patients, prognostication is considered more difficult which was also found within the primary care setting [16, 17, 32, 33]. In patients with organ failure, so-called ‘prognostic paralysis’ can occur, where, because of the uncertainty in prognosis, physicians do not tell patients they have reached the end stage of their disease and do not plan appropriate care [34, 35]. Furthermore, it is well established that physicians experience difficulties in determining prognosis and tend to overestimate life-expectancy [36]. Researchers have suggested that physicians should not wait for a specific prognostic transition point, but instead assess needs to identify the palliative phase [18, 37].

One could argue that weighing of treatment options, another manner for identification described by our respondents, is in fact the consequence of identifying the palliative phase. However, we found that failure or success of treatment determines where professionals believe patients are in their trajectory. This continuous focus on treatment was thought to be stronger in physicians by our respondents, which could be explained by the differences in training. Whereas physicians’ training traditionally focusses on understanding diseases and their cures, nurses are trained in a more holistic approach [38]. Nurses could therefore be considered better assessors of when a patient needs palliative care, which is further supported by the fact that nurses in our study think that patients can more easily open up to them (vs. physicians) about discontinuation of treatment. Yet, supporting previous findings [39], nurses described feeling hesitant to disclose their observations to physicians.

Besides prognostication and treatment-trade-off, our respondents highlighted the importance of appraising patients’ quality of life and holding open conversations with patients and relatives about wishes and preferences. However, not all respondents actively start these conversations and instead wait for initiation by patients. It also seems that the patients’ voice within the assessment of treatment-trade-off is not always taken into account. This finding is not surprising, barriers to starting conversation about the end of life are numerous and divers [31, 40]. One specific barrier described by our respondents is that they did not believe to be the right person to discuss the end of life with patients. Although, one study found that patients think specialists should discuss disease-specific needs and care [15], other studies do bring into question whether hospital-based physicians should be holding these conversations at all [41, 42]. GPs and community nurses, who often have a longer relationship with patients, focus less on a single disease/problem and are aware of functioning at home, might be better positioned to assess needs and consequently identifying the palliative phase. Collaboration between care settings to compare assessments and discuss how to respond to patients’ needs and preferences seems logical. However, only a few physicians in our study described consulting with patients’ GPs. GPs themselves experience this lack of collaboration as an important barrier to improving care for patients at the end of life [33, 43].

### Implications for research and practice

This study indicates that within the hospital setting there is little awareness of the early palliative care model. Both the WHO and the Dutch Quality Framework Palliative Care state that all health care professionals should be aware of the four dimensions of palliative care and early integration is an important aspect [4, 44]. With the raising number of patients in the palliative phase in general hospital departments, efforts should be made to further educate both nurses and physicians on the benefits of early integration of palliative care and how to provide holistic care. Palliative care experts could play an important role in this effort. Furthermore, team trainings have been shown to improve collaboration at hospital departments [45], therefore a combined training on palliative care where both nurses and physicians attend, has the added bonus of improving collaboration on this subject. An important focus of training programs should be on how to initiate conversations with patients about their wishes and preferences, which could result in more patient-centred needs assessment instead of focussing on treatment options and prognosis. Furthermore, collaboration between hospital professionals and primary care professionals needs attention to improve coordination of tasks. What specific barriers exist and how to overcome them, needs further evaluation.

In recent years efforts have been made to provide professionals with instruments to identify patients in the palliative phase. Our respondents felt these identification instruments could potentially be helpful in identification, but doubted the prognostic accuracy. The prognostic accuracy of these instruments indeed varies widely amongst different populations [46, 47]. One could therefore argue whether prognostic value should be the primary objective or whether these instruments should work as a trigger to start conversations about patients’ needs and preferences. Further studies regarding the usability of these identification instruments, and if this indeed leads to earlier integration of palliative care, is needed.

### Strengths and limitations

One of the strengths of this study is that it sampled professionals from both medical and nursing staff, from different department and multiple hospitals. The qualitative approach of this study allowed us to get in-depth insight into each participant’s experiences and helped us gain a better understanding of identification of the palliative phase in daily practise.

The results should be interpreted within their limitations. Although we sampled based on diversity in work backgrounds, we did not sample based on cultural and religious backgrounds—aspects that could influence perspectives on the palliative phase. Furthermore, especially within the sample of medical specialist, many had either training in palliative care or worked within the palliative care team. It might be possible that we therefore did not get the full scope of opinions of less experienced medical specialists. Additionally, IF is a physician herself, her interpretation of the results might carry some bias. We think we have overcome this possible limitation by thoroughly discussing data analysis within our research group.

## Conclusion

Hospital-based physicians and nurses define palliative phase in a variety of ways. Methods used for identification are prognostication, assessment of treatment-trade-off, assessment of needs and preferences and interprofessional collaboration. Practitioners use these means alongside each other, and no structured approach to identification seems to exist. Efforts should be made to create awareness within the hospitals of the early palliative care model and the benefits of timely initiation of palliative care.

## Additional files

Additional file 1: COREQ (COnsolidated criteria for REporting Qualitative research) Checklist. (DOC 63 kb)

Additional file 2: Topic list. (DOCX 16 kb)

## Data Availability

The datasets generated and analysed during the current study are not publicly available due to privacy of respondents, but are available from the corresponding author on reasonable request.

## Abbreviations

COREQ: Consolidated criteria for reporting qualitative research
GP: General practitioner

## References

[1] Davis MP, Temel JS, Balboni T, Glare P (2015). A review of the trials which examine early integration of outpatient and home palliative care for patients with serious illnesses. Ann Palliat Med.

[2] Rabow M, Kvale E, Barbour L, Cassel JB, Cohen S, Jackson V (2013). Moving upstream: a review of the evidence of the impact of outpatient palliative care. J Palliat Med.

[3] Haun MW, Estel S, Rucker G, Friederich HC, Villalobos M, Thomas M (2017). Early palliative care for adults with advanced cancer. Cochrane Database Syst Rev.

[4] World Health Organization (WHO). WHO definition of palliative care. Cited 19 Feb 2019. Available from: https://www.who.int/cancer/palliative/definition/en/.

[5] Lynn Joanne, Adamson David (2003). Living Well at the End of Life: Adapting Health Care to Serious Chronic Illness in Old Age.

[6] Abarshi EA, Echteld MA, Van den Block L, Donker GA, Deliens L, Onwuteaka-Philipsen BD (2011). Recognising patients who will die in the near future: a nationwide study via the Dutch sentinel network of GPs. Br J Gen Pract.

[7] Claessen SJ, Echteld MA, Francke AL, Van den Block L, Donker GA, Deliens L (2012). Important treatment aims at the end of life: a nationwide study among GPs. Br J Gen Pract.

[8] Murray SA, Kendall M, Boyd K, Sheikh A (2005). Illness trajectories and palliative care. BMJ.

[9] Etkind SN, Bone AE, Gomes B, Lovell N, Evans CJ, Higginson IJ (2017). How many people will need palliative care in 2040? Past trends, future projections and implications for services. BMC Med.

[10] Overbeek A, Van den Block L, Korfage IJ, Penders YWH, van der Heide A, Rietjens JAC (2017). Admissions to inpatient care facilities in the last year of life of community-dwelling older people in Europe. Eur J Pub Health.

[11] Barbera L, Taylor C, Dudgeon D (2010). Why do patients with cancer visit the emergency department near the end of life?. CMAJ.

[12] Clark D, Armstrong M, Allan A, Graham F, Carnon A, Isles C (2014). Imminence of death among hospital inpatients: prevalent cohort study. Palliat Med.

[13] Flojstrup M, Henriksen DP, Brabrand M (2017). An acute hospital admission greatly increases one year mortality - getting sick and ending up in hospital is bad for you: a multicentre retrospective cohort study. Eur J Intern Med.

[14] Cotogni Paolo, Saini Andrea, De Luca Anna (2018). In-Hospital Palliative Care: Should We Need to Reconsider What Role Hospitals Should Have in Patients with End-Stage Disease or Advanced Cancer?. Journal of Clinical Medicine.

[15] Beernaert K, Deliens L, De Vleminck A, Devroey D, Pardon K, Van den Block L (2014). Early identification of palliative care needs by family physicians: a qualitative study of barriers and facilitators from the perspective of family physicians, community nurses, and patients. Palliat Med.

[16] Claessen SJ, Francke AL, Engels Y, Deliens L (2013). How do GPs identify a need for palliative care in their patients? An interview study. BMC Fam Pract.

[17] Harrison N, Cavers D, Campbell C, Murray SA (2012). Are UK primary care teams formally identifying patients for palliative care before they die?. Br J Gen Pract.

[18] Dalgaard KM, Bergenholtz H, Nielsen ME, Timm H (2014). Early integration of palliative care in hospitals: a systematic review on methods, barriers, and outcome. Palliat Support Care.

[19] Murray S, Boyd K (2011). Using the 'surprise question' can identify people with advanced heart failure and COPD who would benefit from a palliative care approach. Palliat Med.

[20] Highet G, Crawford D, Murray SA, Boyd K (2014). Development and evaluation of the supportive and palliative care indicators tool (SPICT): a mixed-methods study. BMJ Support Palliat Care.

[21] Thoonsen B, Engels Y, van Rijswijk E, Verhagen S, van Weel C, Groot M (2012). Early identification of palliative care patients in general practice: development of RADboud indicators for PAlliative care needs (RADPAC). Br J Gen Pract.

[22] Malterud K (2001). Qualitative research: standards, challenges, and guidelines. Lancet.

[23] Curry LA, Nembhard IM, Bradley EH (2009). Qualitative and mixed methods provide unique contributions to outcomes research. Circulation.

[24] Tong A, Sainsbury P, Craig J (2007). Consolidated criteria for reporting qualitative research (COREQ): a 32-item checklist for interviews and focus groups. Int J Qual Health Care.

[25] Braun V, Clarke V (2006). Using thematic analysis in psychology. Qual Res Psychol.

[26] MAXQDA. software for qualitative data analysis. VERBI software – consult – Sozialforschung GmbH, Berlin, Germany.1989–2018.

[27] Guest G, Bunce A, Johnson L (2006). How many interviews are enough?. Field Methods.

[28] Shipman C, Gysels M, White P, Worth A, Murray SA, Barclay S (2008). Improving generalist end of life care: national consultation with practitioners, commissioners, academics, and service user groups. BMJ.

[29] Mitchell H, Noble S, Finlay I, Nelson A (2015). Defining the palliative care patient: its challenges and implications for service delivery. BMJ Support Palliat Care.

[30] Van Mechelen W, Aertgeerts B, De Ceulaer K, Thoonsen B, Vermandere M, Warmenhoven F (2013). Defining the palliative care patient: a systematic review. Palliat Med.

[31] Horlait M, Chambaere K, Pardon K, Deliens L, Van Belle S (2016). What are the barriers faced by medical oncologists in initiating discussion of palliative care? A qualitative study in Flanders, Belgium. Support Care Cancer.

[32] Zheng L, Finucane A, Oxenham D, McLoughlin P, McCutcheon H, Murray S (2013). How good is primary care at identifying patients who need palliative care? A mixed methods study. Eur J Palliat Care.

[33] Wichmann AB, van Dam H, Thoonsen B, Boer TA, Engels Y, Groenewoud AS (2018). Advance care planning conversations with palliative patients: looking through the GP’s eyes. BMC Fam Pract.

[34] Gadoud A, Kane E, Macleod U, Ansell P, Oliver S, Johnson M (2014). Palliative care among heart failure patients in primary care: a comparison to cancer patients using English family practice data. PLoS One.

[35] Stewart S, McMurray JJV (2002). Palliative care for heart failure : time to move beyond treating and curing to improving the end of life. BMJ.

[36] White N, Reid F, Harris A, Harries P, Stone P (2016). A systematic review of predictions of survival in palliative care: how accurate are clinicians and who are the experts?. PLoS One.

[37] Kendall M, Buckingham S, Ferguson S, Hewitt N, Pinnock H (2014). We need to stop looking for something that is not there. NPJ Prim Care Respir Med.

[38] Tang CJ, Chan SW, Zhou WT, Liaw SY (2013). Collaboration between hospital physicians and nurses: an integrated literature review. Int Nurs Rev.

[39] Gott M, Ingleton C, Bennett MI, Gardiner C (2011). Transitions to palliative care in acute hospitals in England: qualitative study. BMJ Support Palliat Care.

[40] Tavares Nuno, Jarrett Nikki, Hunt Katherine, Wilkinson Tom (2017). Palliative and end-of-life care conversations in COPD: a systematic literature review. ERJ Open Research.

[41] Lovell A, Yates P (2014). Advance care planning in palliative care: a systematic literature review of the contextual factors influencing its uptake 2008-2012. Palliat Med.

[42] Seamark D, Blake S, Seamark C, Hyland ME, Greaves C, Pinnuck M (2012). Is hospitalisation for COPD an opportunity for advance care planning? A qualitative study. Prim Care Respir J.

[43] Oosterink JJ, Oosterveld-Vlug MG, Glaudemans JJ, Pasman HR, Willems DL, Onwuteaka-Philipsen BD (2016). Interprofessional communication between oncologic specialists and general practitioners on end-of-life issues needs improvement. Fam Pract.

[44] IKNL/Palliactief (2017). Kwaliteitskader palliatieve zorg Nederland.

[45] Weaver SJ, Dy SM, Rosen MA (2014). Team-training in healthcare: a narrative synthesis of the literature. BMJ Qual Saf.

[46] Downar J, Goldman R, Pinto R, Englesakis M, Adhikari NK (2017). The “surprise question” for predicting death in seriously ill patients: a systematic review and meta-analysis. CMAJ.

[47] De Bock R, Van Den Noortgate N, Piers R (2018). Validation of the supportive and palliative care indicators tool in a geriatric population. J Palliat Med.

[48] Medical Research (Human Subjects) Act 2017 Available from: https://english.ccmo.nl/investigators/legal-framework-for-medical-scientific-research/laws/medical-research-involving-human-subjects-act-wmo.

