# Supplementary material for: How do hospital-based nurses and physicians identify the palliative phase in their patients and what difficulties exist? A qualitative interview study
Source: BMC Palliat Care. 2019 Jul 9;18:54. doi: 10.1186/s12904-019-0439-0 (PMC6617645; doi:10.1186/s12904-019-0439-0)
Supplement: Supplementary file 2 — Topic list. (DOCX 16 kb) [file 12904_2019_439_MOESM2_ESM.docx]

Appendix 2. Topic list

General

- Can you tell me about the last patient you thought was in the palliative phase?

-What made you think this patient was in the palliative phase?

-How do you define the palliative phase?

-Can you tell me about a patient where you doubt if he or she is in the palliative phase?

-What makes you doubt?

-Can you tell me about a case where you were to early/late in identifying the palliative phase?

-Are there consequences when u identify the palliative phase?

Difficulties

-What makes identification of the palliative phase easy/difficult?

-Are there differences in identification for different diseases?

Identification instruments

-Have you heard of instruments that can aid in identification of the palliative phase? If yes, do you use them?
